# Hypoxia Delays Oligodendrocyte Progenitor Cell Migration and Myelin Formation by Suppressing Bmp2b Signaling in Larval Zebrafish

**DOI:** 10.3389/fncel.2018.00348

**Published:** 2018-10-04

**Authors:** Lei-qing Yang, Min Chen, Jun-long Zhang, Da-long Ren, Bing Hu

**Affiliations:** ^1^Hefei National Laboratory for Physical Sciences at the Microscale, School of Life Sciences, University of Science and Technology of China, Hefei, China; ^2^Chinese Academy of Sciences Key Laboratory of Brain Function and Disease, School of Life Sciences, University of Science and Technology of China, Hefei, China

**Keywords:** hypoxia, oligodendrocyte, differentiation, myelination, bmp2b, zebrafish

## Abstract

Hypoxia in newborns tends to result in developmental deficiencies in the white matter of the brain. As previous studies of the effects of hypoxia on neuronal development in rodents and human infants have been unable to use *in vivo* imaging, insight into the dynamic development of oligodendrocytes (OLs) in the central nervous system under hypoxia is limited. Here, we developed a visual model to study OL development using sublethal postnatal hypoxia in zebrafish larvae. We observed that hypoxia significantly suppressed OL progenitor cell migration toward the dorsum using *in vivo* imaging. Further, we found that hypoxia affected myelination, as indicated by thinner myelin sheaths and by a downregulation of myelin basic protein expression. Bmp2b protein expression was also significantly downregulated following hypoxia onset. Using gain of function and loss of function experiments, we demonstrated that the Bmp2b protein was associated with the regulation of OL development. Thus, our work provides a visual hypoxia model within which to observe OL development *in vivo*, and reveals the underlying mechanisms involved in these processes.

## Introduction

Prior clinical research has indicated that adequate oxygen delivery is vital for brain development in infants (Hack et al., 2000; Back et al., 2006; Chahboune et al., 2009). Many investigators have modeled clinical symptoms in experimental animals by exposing neonatal rodents to different degrees and durations of hypoxia (Ganat et al., 2002; Kanaan et al., 2006; Zhou et al., 2008; Fagel et al., 2009). In both mice and rats, hypoxia reduces the volumes of the cerebral cortex and corpus callosum, and eventually leads to liberal ventriculomegaly (Weiss et al., 2004; Watzlawik et al., 2015). Hypoxia also has been shown to disrupt synaptic development (Curristin et al., 2002), binuclear neuron formation (Paltsyn et al., 2014), and glia–neuron interactions (Accorsi-Mendonca et al., 2015). Thus, hypoxia presumably abrogates the development and cognitive potential of the newborn brain (Huppi et al., 2001; Sabatino et al., 2003). A recent study demonstrated that targeting EGFR in oligodendrocyte (OL) progenitor cells (OPCs) at a specific time after premature newborn hypoxic injury was clinically feasible and potentially beneficial (Scafidi et al., 2014). Thus, it is clear that hypoxia plays an important role in the development of neurons and glia in the brain. However, neural behavior, especially OL behavior, in the spinal cord has rarely been monitored *in vivo* in conjunction with hypoxia.

Zebrafish (*Danio rerio)* represent an attractive vertebrate model for developmental research due to their rapid development (Kimmel et al., 1995) and transparency, allowing easy *in vivo* imaging. Moreover, as mammalian and zebrafish OLs and myelin are homologous, the transparent zebrafish model represents an efficient tool for the observation of OL differentiation and myelination; using zebrafish, it is also possible to explore molecular mechanisms *in vivo* via a number of previously described genetic manipulations and fluorescence transgeneses (Buckley et al., 2008; Dubois-Dalcq et al., 2008; He et al., 2014). Although hypoxia-induced retinopathy (Cao et al., 2010), bone regeneration (Maes et al., 2012), and cancer metastasis (Kumar and Gabrilovich, 2014) have been widely studied, little is known about the mechanism of OL development in the hypoxic zebrafish model.

Here, we found that hypoxia inhibited OPC dorsal migration, delayed the onset of OL myelination *in vivo*, and reduced the expression of the *mbp* gene. Transmission electron microscope (TEM) images showed a thinner myelin sheath in response to hypoxic conditions. We used molecular methods to show that the *bmp2b* gene was downregulated under hypoxia. Using a Bmp2b receptor inhibitor and *bmp2b* messenger RNA (mRNA) rescue strategy, we demonstrated that Bmp2b participated in regulating OL development under hypoxia. Collectively, our findings indicated that hypoxia suppresses OL differentiation via Bmp2b signaling.

## Materials and Methods

### Zebrafish Lines and Maintenance

The following zebrafish lines were used in this study: wild-type (WT), transgenic OL lineage transcription factor 2 labeled by EGFP [Tg (*olig2*:EGFP)] (Shin et al., 2003), Tg (mbp:EGFP-CAAX; donated by Prof. David A. Lyons, University of Edinburgh, Edinburgh, United Kingdom) (Almeida et al., 2011) and Tg (Tol-056) (Satou et al., 2009). Zebrafish embryos were bred with laboratory stock and were maintained at 28.5°C with a 14/10 h light/dark cycle. Zebrafish embryos were collected from natural spawning and staged by days post-fertilization (dpf), according to established criteria (Kimmel et al., 1995). To prevent dark pigment formation, larvae were raised in an embryo medium containing 0.2 mM *N*-phenylthiourea (Sigma-Aldrich, St. Louis, MO, United States).

All of the animal manipulations described in this study were conducted in strict accordance with the guidelines and regulations set forth by the University of Science and Technology of China (USTC) Animal Resources Center and University Animal Care and Use Committee. All protocols were approved by the Committee on the Ethics of Animal Experiments of the USTC (permit no. USTCACUC1103013).

### Hypoxia Device

The hypoxia device we used contained a self-driven unit that automatically controlled nitrogen gas perfusion depending on dissolved oxygen levels in the water (Cao et al., 2010). An oxygen electrode was connected to an oxygen regulator and to the nitrogen gas supplier to accurately detect oxygen levels continuously. We used 100% of the ambient oxygen in the air (7.10 ppm) as the calibration concentration, recalibrated to 40% (3.41 ppm) based preliminary results. To reduce the likelihood of unexpected early zebrafish death during experimentation, the oxygen regulator was recalibrated before each experiment.

### *In vivo* Imaging and Data Analysis

Larvae to be imaged were transferred to *N*-phenylthiourea to inhibit pigmentation at 24 h post-fertilization (hpf). Prior to imaging, larvae were anesthetized with MS222 and embedded in 1% low-melting-point agarose in embryo medium containing MS222. All images of the spinal cord were taken laterally, such that anterior was to the left, and dorsal was at the top. To investigate OPC migration at 3 and 4 dpf *in vivo*, Tg (*olig2*: EGFP) transgenic zebrafishes were imaged under a 10 × objective lens and a 40 × objective lens (FV1000 BX61; Olympus, Tokyo, Japan) at 2 μm intervals, locating within a frame long and wide with the cloacal pores at the central point of the spinal cord. After imaging, fish were removed from the agarose and placed in fresh embryo medium for further growth. Photomontages were assembled with Adobe Photoshop CS5 (Adobe Systems, San Jose, CA, United States).

### Treatment With CoCl_2_⋅6H_2_O and LDN193189

Cobalt (II) chloride hexahydrate (CoCl_2_⋅6H_2_O; Sangon, China) is a general drug designed to induce hypoxia *in vitro* (Wu et al., 2015). LDN193189 (S2618; Selleck Chemicals, United States) was used as a Bmp2b receptor inhibitor. We treated 1-dpf larvae with either 2 mM CoCl_2_⋅6H_2_O or 10 μM LDN193189 in subsequent experiments.

### Quantitative Real-Time Polymerase Chain Reaction (qRT-PCR) and Western Blots

Total mRNA was extracted from three groups of 30 larvae each, with each group representing a single independent sample. Each sample was reverse-transcribed into complementary DNA (cDNA) with HiScriptII Q RT SuperMix (Vazyme, China), and analyzed with qRT-PCR using AceQ qPCR SYBR Green Master Mix (Vazyme, China). For each experiment, we used three biological and experimental replicates. All primers used are given in **Supplementary Table S1**. All results were analyzed as mean fold change ± SEM.

To detect protein expression of Mbp, Bmp2b, and Hif1α in zebrafish larvae, we collected normoxic and hypoxic WT zebrafish larvae at 3 and 4 dpf. Larvae were lysed with RIPA buffer (Beyotime, China). Equal amounts of pooled normoxic and hypoxic extracts were loaded onto SDS–PAGE gels. Western blots were performed using the antibodies to Mbp (1:1,000; Abmart, Shanghai, China), Bmp2b (1: 1,000; GTX54233; Genetex, Irvine, CA, United States), HIF1α (1:500; NB100-134; Novus), and β-actin (1:1,000; GTX629630; Genetex, Irvine, CA, United States) as primary antibodies. The MBP rabbit polyclonal antibody was generated (by Abmart, Shanghai, China) using the peptide SRSRSPPKRWSTIF as previously described (Lyons et al., 2005). The HRP-conjugated goat anti-rabbit secondary antibody (Invitrogen, Carlsbad, CA, United States) was diluted to 1: 10,000 at room temperature.

### *Bmp2b* mRNA Synthesis and Microinjection

To generate *bmp2b* mRNA, a full-length *bmp2b* fragment was initially amplified from the cDNA of the WT zebrafish strain using a pair of primers (**Supplementary Table S1**). *bmp2b* mRNA was then synthesized with a T7 mMESSAGE mMACHINE Kit (Ambion, Foster City, CA, United States). Finally, we injected 200 pg *bmp2b* mRNA into single-cell zebrafish embryos.

### Zebrafish Optokinetic Response (OKR) Assays

Optokinetic Response assays were performed as previously described (Mueller and Neuhauss, 2010; Huang et al., 2018). We used these assays to visually examine the functional deficiencies of 4-dpf hypoxic zebrafish larvae after OL development disruption. We used LabVIEW to generate a sine-wave grating and used an LCD projector to project this grating. Zebrafish larvae were immobilized dorsal side up in 6% methylcellulose in a chamber. Elicited eye movements were recorded in real time by an infrared-sensitive CCD camera (TCA-1.3BW; Nanjing, China), while rotating grating patterns were projected around the larva. Normoxic and hypoxic larvae were stimulated at a constant angular velocity of 7.5°. To measure visual acuity, both sets of larvae were presented with spatial frequencies of 0.02, 0.04, and 0.06 cycles/degree at 3, 6, and 9 continuous contrast levels, respectively. The OKR gain (the ratio of eye velocity to stimulus velocity) was used to measure contrast sensitivity (Rinner et al., 2005).

### Transmission Electron Microscopy

All larval zebrafish were immobilized overnight in a 2.0% formaldehyde and 2.5% glutaraldehyde fixative solution (Electron Microscopy Sciences, United States) at 4°C. Immobilized larval zebrafish were washed with 0.1 M phosphate buffer (pH 7.4). Specimens were then incubated in a postfixation solution containing 1% osmium tetroxide for 2 h and washed with water. Specimens were then washed three times with 0.1 M phosphate buffer (pH 7.4) for 15 min each time. Next, specimens were washed with water, and twice dehydrated with serial dilutions of ethanol in water (i.e., 50, 70, 80, 90, and 100%) and with 100% for 15 min each. All samples were then embedded in epon/araldite resin and hardened for 2 to 3 days at 60°C. Ultrathin (80 nm) transverse sections of the spinal cords of all larvae were stained with uranyl acetate and lead citrate. Sections were viewed and photographed with an FEI Tecnai Spirit (120 kV TEM) transmission electron microscope.

### Statistical Analysis

Values were presented as means ± SEMs. The significance of differences between or among groups were identified using Student’s *t*-tests, non-parametric tests, one-way analyses of variance (ANOVA), or two-way ANOVAs, based on the number of groups compared and other independent factors. For qRT-PCR analyses, we analyzed three independent samples three times each to yield reliable results. Statistical significance was classed as follows: ^∗^*P* < 0.05, ^∗∗^*P* < 0.01, and ^∗∗∗^*P* < 0.001.

## Results

### Creation of Moderate Sublethal Hypoxic Conditions for Larval Zebrafish

**FIGURE 1:**
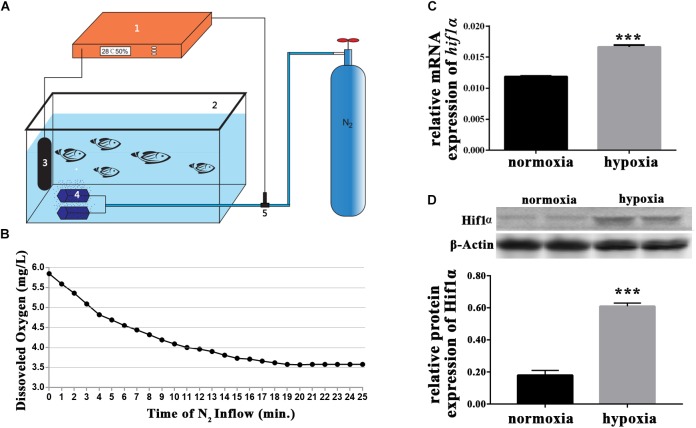
Hypoxic setup for larval zebrafish. **(A)** (1) oxygen regulator; (2) hypoxic larval aquarium; (3) oxygen electrode; (4) air stone; and (5) solenoid valve. The compressed nitrogen source is shown as a blue gas cylinder. **(B)** Dissolved oxygen in the hypoxic aquarium decreased slowly over time as nitrogen perfusion increased until dissolved oxygen stabilized at 3.5 mg/L. **(C)** qRT-PCR analysis showing that *hif1α* mRNA expression was significantly increased under hypoxia. **(D)** Western blot of Hif1α protein expression showing that hypoxia enhanced Mbp protein translation. ^∗^*P* < 0.05; ^∗∗^*P* < 0.01; ^∗∗∗^*P* < 0.001.

To create hypoxic conditions for larval zebrafish, nitrogen gas was perfused into the water of a 1 L aquarium. The aquarium was an automated unit (**Figure 1A**). Oxygen levels were accurately measured by the oxygen electrode, which was connected to the oxygen regulator and the nitrogen gas supplier. Thus, nitrogen gas perfusion was controlled depending on the levels of dissolved oxygen in the water. Preliminary studies indicated that dissolved oxygen levels of 3.5 mg/L were optimal for our experiments (**Figure 1B**). Preliminary experiments also showed that the gene expression of *hif1α* and the protein expression of Hif1*α* increased in response to hypoxia (**Figures 1C,D**). Thus, we successfully created sublethal hypoxic conditions by automatically regulating the perfusion of nitrogen into the water.

### Hypoxia Suppressed OL Differentiation

**FIGURE 2:**
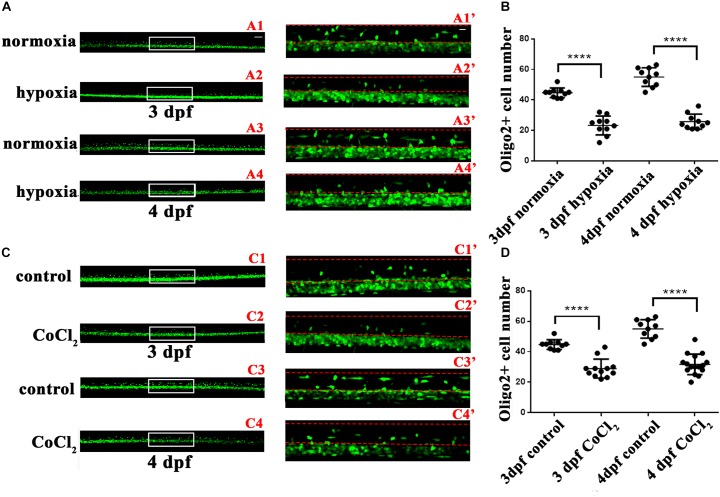
Hypoxia and CoCl_2_⋅6H_2_O suppressed OPC migration to the dorsal area. The number of dorsally migrated spinal cord *olig2^+^* cells was counted in Tg (*olig2*: EGFP) zebrafish using a 10 × objective lens on a FV1000 microscope (Olympus, Tokyo, Japan). **(A,B)** The number of dorsally migrated *olig2^+^* cells decreased under hypoxia at 3 and 4 dpf. A1′, A2′, A3′, and A4′ are the magnified pictures of the white boxes in A1, A2, A3, and A4. Two-way ANOVA, *P* < 0.0001: Student’s two-tailed *t*-test. Significantly more cells were dorsally migrated in 3-dpf normoxic larvae as compared to 3-dpf hypoxic larvae (*n* = 10; *P* < 0.0001). Significantly more cells were dorsally migrated in 4-dpf normoxic larvae as compared to 4-dpf hypoxic larvae (*n* = 10; *P* < 0.0001). **(C,D)** The number of dorsally migrated spinal cord *olig2^+^* cells in 3- and 4-dpf larvae decreased after CoCl_2_⋅6H_2_O exposure. C1′, C2′, C3′, and C4′ are the magnified pictures of the white boxes in C1, C2, C3, and C4. Two-way ANOVA, *P* < 0.0001: Student’s two-tailed *t*-test. Significantly more cells were dorsally migrated in 3-dpf normoxic larvae as compared to 3-dpf hypoxic larvae (*n* = 10; *P* < 0.0001). Significantly more cells were dorsally migrated in 4-dpf normoxic larvae as compared to 4-dpf hypoxic larvae (*n* = 10; *P* < 0.0001). The dashed line indicates the dorsal spinal cord. Scale bars: A1–A4 and C1–C4, 50 μm; A1′–A4′, and C1′–C4′, 10 μm. ^∗^*P <* 0.05; ^∗∗^*P <* 0.01; ^∗∗∗^*P <* 0.001. Error bars represent S.E.M.

In infants with periventricular leukomalacia, the delivery of oxygen to the developing brain often fails; in addition, these infants also exhibit widespread changes in glial maturation, with a loss of myelinated fiber tracts (Haynes et al., 2012). To investigate the OL changes that might occur in the central nervous system *in vivo* in response to hypoxia, we first observed OPC migration in the Tg (*olig2*: EGFP) transgenic line. At 24 hpf, embryos subjected to hypoxia, and larvae were collected for living imaging at 3 and 4 dpf. At both 3 and 4 dpf, significantly fewer dorsally migrated spinal cord *olig2^+^* cells were observed in the hypoxic larvae (*n* = 10; 3 dpf: 23.30 ± 1.955; 4 dpf: 25.80 ± 1.562) (**Figure 2B**) as compared to the normoxic larvae (*n* = 10; 3 dpf: 44.70 ± 1.012; 4 dpf: 55.00 ± 1.921; **Figure 2A**).

To further test these results, we used cobalt chloride (CoCl_2_) to induce similar hypoxia conditions (Goldberg et al., 1988; Wu et al., 2015). At 24 hpf, 2 mM of CoCl_2_⋅6H_2_O (Sangon, China) was used to mimic hypoxia. We then collected larvae at 3 and 4 dpf to observe OPC migration. We observed far fewer dorsally migrated spinal cord *olig2^+^* cells in the CoCl_2_⋅6H_2_O group (*n* = 10; 3 dpf: 28.92 ± 1.803; 4 dpf: 31.69 ± 1.685) than in the control group (*n* = 10; 3 dpf: 44.70 ± 1.012; 4 dpf: 55.00 ± 1.921; **Figures 2C,D**). This result suggested that some *olig2^+^* cells differentiated and matured early, beginning functional myelination locally, and were thus unable to migrate to their normal destinations. Together, these results indicated that hypoxia suppresses zebrafish OPC differentiation *in vivo*.

### Hypoxia Induced Delayed OL Myelination

**FIGURE 3:**
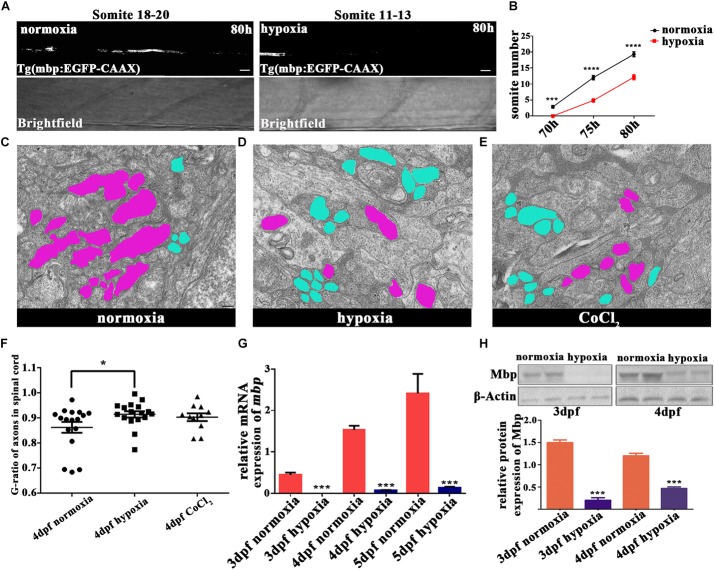
Hypoxia delayed the onset of myelination and led to thinner myelin sheaths. **(A)** Myelination of the Mauthner axon, the most posterior myelin sheath in the spinal cord, at 80 hpf. Myelination can be seen between somites 18 and 20 in normoxic animals but between somites 11 and 13 in hypoxic animals. Scale bars: 10 μm. **(B)** The most posterior myelin sheath at 70 hpf is somite 2.875 ± 0.3981 in normoxic animals (*n* = 8) and somite 0.0 ± 0.0 in hypoxic animals (*n* = 8); at 75 hpf is somite 12.00 ± 0.6814 in normoxic animals (*n* = 8) and somite 4.857 ± 0.5084 in hypoxic animals (*n* = 7); and at 80 hpf is somite 19.27 ± 0.7519 in normoxic animals (*n* = 8) and somite 12.13 ± 0.7892 in hypoxic animals (*n* = 7). Two-way ANOVA, *P* < 0.0001. **(C–E)** A large number of unmyelinated axons (shaded in blue) were observed in the spinal cord axons of the hypoxic and CoCl_2_ groups as compared to the myelinated axons (shaded in purple) observed in the normoxic group. Scale bars: 200 nm. **(F)** The hypoxic group had a higher G-ratio in the spinal cord axon than did the normoxic group, indicating delayed-onset myelination under hypoxia. One-way ANOVA, *P <* 0.05. **(G)** qRT-PCR analysis indicated that myelin basic protein (*mbp*) mRNA transcription was significantly decreased under hypoxia. **(H)** The expression of the Mbp protein was analyzed with western blots. The results showed that hypoxia blocked Mbp protein translation. Larvae at 3 and 4 dpf (30 per day) were pooled for western blot experiments. ^∗^*P* < 0.05; ^∗∗^*P* < 0.01; ^∗∗∗^*P* < 0.001; ^∗∗∗∗^*P* < 0.0001.

To explore the subsequent OL developmental processes, we observed mature OLs. Myelination occurs along an anteroposterior gradient in the developing spinal cord (Buckley et al., 2008). Therefore, we used the timings of the appearance of myelin sheaths at specific sites along the anteroposterior axis as the indices of myelination development. For this experiment, we used Tg (mbp:EGFP-CAAX) zebrafish, and found that the onset of myelination was delayed under hypoxia (**Figures 3A,B**). In normoxic zebrafish larvae, the onset of myelination was 3 dpf, which coincided with *mbp* gene transcription and dorsal OL migration/differentiation (Buckley et al., 2010).

To further evaluate myelination under hypoxia, we used electron microscopy to examine the myelination of zebrafish axons in the spinal cord at 4 dpf (**Figures 3C–E** and **Supplementary Figure S2**). We found that many more axons were myelinated than unmyelinated in the normoxic group, whereas the opposite result was observed in the hypoxic and CoCl_2_ groups. We then used the G-ratio (the axon circumference divided by the total circumference) to compare the number of myelinated axons between groups (Yin and Hu, 2014). The axon G-ratios in the hypoxic groups (*n* = 17; 0.9144 ± 0.01243) and in the CoCl_2_ groups (*n* = 11; 0.9029 ± 0.01559) were also higher than those of the normoxic groups (*n* = 17; 0.8623 ± 0.02167) (**Figure 3F**).

We further investigated the effects of hypoxia on OPC migration and axonal myelination by quantifying the gene expression of *mbp* and *olig2* and the protein expression of Mbp in hypoxic zebrafish larvae at 3 dpf and 4 dpf. Our qRT-PCR results showed that at 3, 4, and 5 dpf, *mbp* and *olig2* mRNA expression were significantly lower in hypoxic zebrafish larvae as compared to normoxic larvae (**Figure 3G** and **Supplementary Figure S1**). The protein expression of Mbp was also substantially lower in the hypoxia group as compared to the normoxic group (**Figure 3H**).

Using OKR to assess the function of the visual system, previous studies have also demonstrated that OL affects OKR in zebrafish (Yin and Hu, 2014; Tian et al., 2016). Here, our OKR test revealed that, under hypoxia, central nervous system myelination was delayed and that myelin sheaths were more thinly wrapped. Additionally, our OKR experiment indicated that visual function was also deficient in 4-dpf hypoxic zebrafish (**Supplementary Figure S3**).

Thus, our results indicated that hypoxia disrupts OL development *in vivo*, both in terms of OPC migration and with respect to mature OL myelination.

### LDN193189 Inhibited OPC Dorsal Migration

**FIGURE 4:**
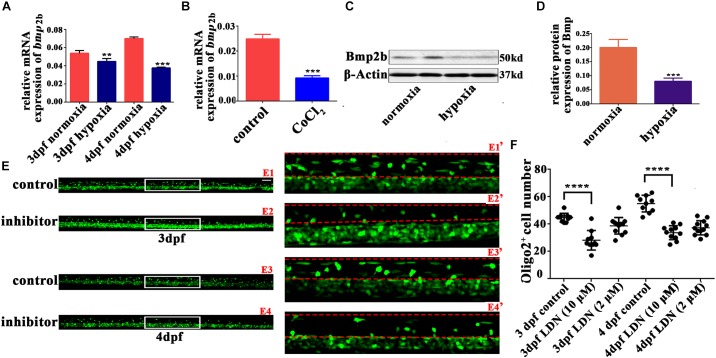
The Bmp2b inhibitor suppresses OPC migration to the dorsal area. **(A,B)** qRT-PCR analysis indicated that *bmp2b* gene mRNA expression was significantly downregulated after hypoxia and CoCl_2_ exposure. **(C,D)** Bmp2b protein expression was analyzed with western blots. The results showed that hypoxia blocked Bmp2b protein translation. Thirty zebrafish 4-dpf larvae were pooled for western blots experiments. **(E,F)** The number of dorsally migrated *olig2^+^* cells at 3 and 4 dpf decreased after LDN193189 treatment. E1′, E2′, E3′, and E4′ are magnified pictures of the white boxes in E1, E2, E3, and E4. Scale bars: E1–E4, 50 μm and E1′–E4′, 10 μm. Two-way ANOVA, *P* < 0.0001: Student’s two-tailed *t*-test. Significantly more cells were dorsally migrated in 3-dpf control larvae as compared to 3-dpf larvae treated with 10 μM LDN193189 (*n* = 12; *P* < 0.0001). Significantly more cells were dorsally migrated in 4-dpf normoxic larvae as compared to 4-dpf larvae treated with 10 μM LDN193189 (*n* = 10; *P* < 0.0001). ^∗^*P <* 0.05; ^∗∗^*P <* 0.01; ^∗∗∗^*P <* 0.001; ^∗∗∗∗^*P <* 0.0001. Error bars represent S.E.M.

BMP deficiency affects glial maturation in the spinal cord (See et al., 2007). BMPs also act as growth factors, stimulating axon growth in the spinal motor neurons (Zhong and Zou, 2014). Bmp2b and Bmp4 are two classic BMP isoforms that regulate the early development of zebrafish (Grinspan et al., 2000; Mowbray et al., 2001; Ke et al., 2008; Tiso et al., 2009). Therefore, we measured the gene expression of *bmp2b* and *bmp4* with qRT-PCR. We found that *bmp2b* gene expression was strongly downregulated in 3 and 4 dpf hypoxic zebrafish larvae vs. normoxic larvae (**Figures 4A,B**); there was no apparent change *bmp4* gene expression (data not shown). Bmp2b protein expression was also obviously downregulated in the hypoxic larvae as compared to the normoxic larvae (**Figures 4C,D**).

We next used a Bmp2b receptor inhibitor to observe OPC migration, and found that significantly fewer spinal cord *olig2^+^* cells were dorsally migrated in the larvae treated with 10 μM LDN193189 at 3 dpf (*n* = 10; 28.20 ± 2.274) and 4 dpf (*n* = 11; 33.73 ± 1.544), as compared to the untreated larvae at 3 dpf (*n* = 10; 44.70 ± 1.012) and 4 dpf (*n* = 10; 55.00 ± 1.921; **Figures 4E,F**). These results suggested that Bmp2b might play a pivotal role in the regulation of OPC differentiation under hypoxia.

### *Bmp2b* mRNA Reversed the Abnormalities in OPC Differentiation

**FIGURE 5:**
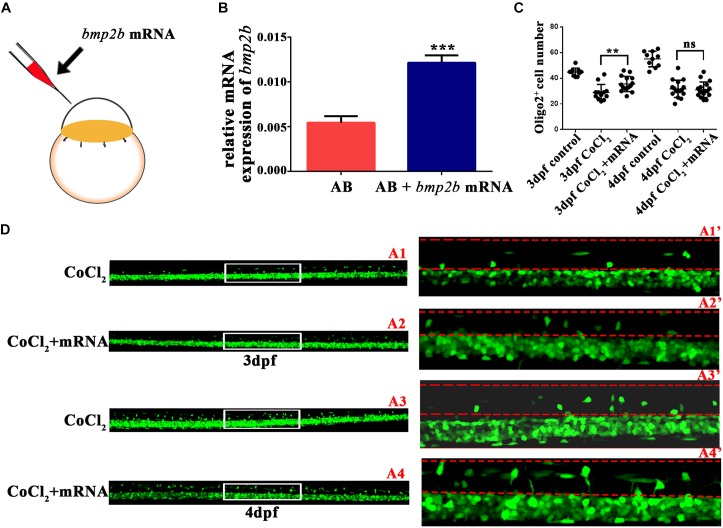
*Bmp2b* mRNA reversed the reduction in the number of *olig2^+^* cells migrating to the dorsal area. **(A)** *Bmp2b* mRNA was injected into one-cell embryos. **(B)** qRT-PCR analysis showed that *bmp2b* gene expression increased, validating the rescue effect. **(C–D)** *bmp2b* mRNA reversed the reduction in the number of dorsally migrated *olig2^+^* cells at 3 and 4 dpf. A1′, A2′, A3′, and A4′ are the magnified pictures of the white boxes in A1, A2, A3, and A4. Scale bars: A1–A4, 50 μm and A1′–A4′, 10 μm. ^∗^*P <* 0.05; ^∗∗^*P <* 0.01; ^∗∗∗^*P <* 0.001. Error bars represent S.E.M.

To investigate whether *bmp2b* was essential to OPC migration under hypoxia, we synthesized *bmp2b* mRNA and injected it into one-cell Tg (*olig2*: EGFP) zebrafish embryos. We then measured *bmp2b* gene expression at 3 dpf (**Figures 5A,B**). We found that *bmp2b* mRNA injection after CoCl_2_ increased the dorsally migrated spinal cord *olig2^+^* cells at 3 dpf (*n* = 15; 35.53 ± 1.521) and 4 dpf (*n* = 17; 31.06 ± 1.459), as compared to CoCl_2_-treated larvae not injected with *bmp2b* mRNA at 3 dpf (*n* = 12; 28.92 ± 1.803) and 4 dpf (*n* = 16; 31.69 ± 1.685; **Figures 5C,D**). These results indicated that Bmp2b signaling is critical to OPC differentiation.

## Discussion

Hypoxia is a principal cause of brain injury in premature infants, which clinical features consist of diagnostic aspects and clinicopathologic correlations (du Plessis, 2009). Using a hypoxic larval zebrafish model, we identified several novel aspects of OL behavior in the spinal cord under hypoxia. First, we used Tg (*olig2*: EGFP) and Tg (mbp-EGFP-CAAX) hypoxic models to show that fewer OPCs migrated to the dorsal area and that myelin sheaths in the spinal cord were thinner. Second, *bmp2b* gene expression was downregulated in hypoxic animals. Third, based on loss of function and gain of function, we demonstrated that hypoxia suppressed OL differentiation through Bmp2b signaling.

Hypoxia affects many processes involved in bone regeneration (Drager et al., 2015) and angiogenesis (Tanaka and Nangaku, 2013). Several rodent models have been designed to study various hypoxia-induced diseases, including retinopathy (Liu et al., 2014), bronchopulmonary dysplasia (Elberson et al., 2015), and absolutely periventricular leukomalacia (Chen et al., 2015). However, the zebrafish has become an attractive vertebrate model organism for the study of hypoxia-induced diseases due to the broad conservation of its genes and its bodily transparency (Cao et al., 2010; Wu et al., 2015). Here, we observed OPC migration and OL myelination *in vivo.* We found that, under hypoxia, significantly fewer *olig2^+^* cells migrated to the dorsal area, and *olig2* gene expression was downregulated. The onset of myelination was also delayed under hypoxia, which may have been due to changes in *mbp* transcription and dorsal OL migration and differentiation (Buckley et al., 2010). We used TEM to show that spinal cord axons were covered with a thinner myelin sheath under hypoxia, indicating that hypoxia severely disrupted OL function. Our results thus indicated that hypoxia affected OPC migration, OL myelination, and even myelin sheath thickness.

We also investigated the effects of Bmp2b on OL differentiation under hypoxia using loss of function and gain of function. Consistent with our results, BMPs have been shown to be associated with OPC differentiation (See et al., 2007). Although the mechanisms by which hypoxia affects Bmp2b expression, several studies have shown that BMPs are associated with OL differentiation, and that hypoxia affects BMP2protein expression (Drager et al., 2015). Thus, we speculated that Bmp2b might be involved in reducing OL differentiation in zebrafish. However, our results do not exclude the possibility that additional genes or BMP subunits may also be regulated by hypoxia.

To test our speculation, we designed *bmp2b* rescue mRNA. For the OPC migration experiment, we obtained the desired rescue effect by injecting *bmp2b* mRNA into one-cell-phase embryos.

## Conclusion

In summary, our results indicated that hypoxia negatively influences OL differentiation, and that this process was meditated by Bmp2b signaling. Our ultimately increases our understanding of hypoxia and glial development.

## Author Contributions

L-qY, D-lR, and BH designed the experiments. L-qY conducted all the experiments and wrote the manuscript. MC and J-lZ also helped to complete part of the experiments. BH and D-lR revised the manuscript.

## Conflict of Interest Statement

The authors declare that the research was conducted in the absence of any commercial or financial relationships that could be construed as a potential conflict of interest.
