# Supplementary material for: Hypoxia Delays Oligodendrocyte Progenitor Cell Migration and Myelin Formation by Suppressing Bmp2b Signaling in Larval Zebrafish
Source: Front Cell Neurosci. 2018 Oct 4;12:348. doi: 10.3389/fncel.2018.00348 (PMC6180284; doi:10.3389/fncel.2018.00348)
Supplement: Supplementary file 1 [file Data_Sheet_1.PDF]

Supplementary Material

Hypoxia delays oligodendrocyte progenitor cell migration and myelin formation through suppression of Bmp2b signaling in larval zebrafish

Lei-qing Yang[1], Min Chen[1], Jun-long Zhang[1], Da-long Ren[1*], Bing Hu[1,2*]

[1]Hefei National Laboratory for Physical Sciences at the Microscale, School of Life Sciences, University of Science and Technology of China, Hefei 230027, China;

[2]Chinese Academy of Sciences Key Laboratory of Brain Function and Disease, School of Life Sciences, University of Science and Technology of China, No. 96 Jinzhai Road, Hefei, Anhui Province, 230026, P. R. China.

Office 86-551-63602489

Lab 86-551-63607014

Fax 86-551-63601443

*Correspondence author: Da-long Ren rendl@ustc.edu.cn

Bing Hu bhu@ustc.edu.cn

1 Supplementary Figures and Tables

1.1 Supplementary Figure 1

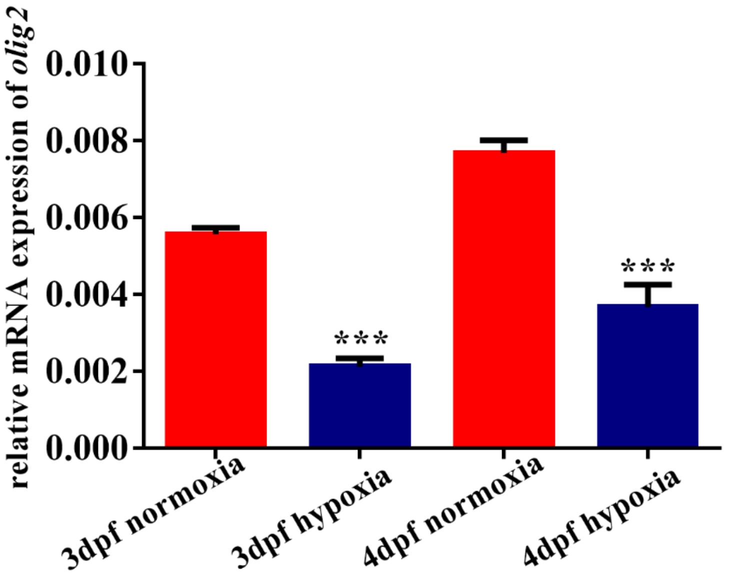

Supplementary Figure 1. qRT-PCR analysis revealed that *olig2* gene mRNA levels were significantly decreased with hypoxia at 3 and 4 dpf respectively.

1.2 Supplementary Figure 2

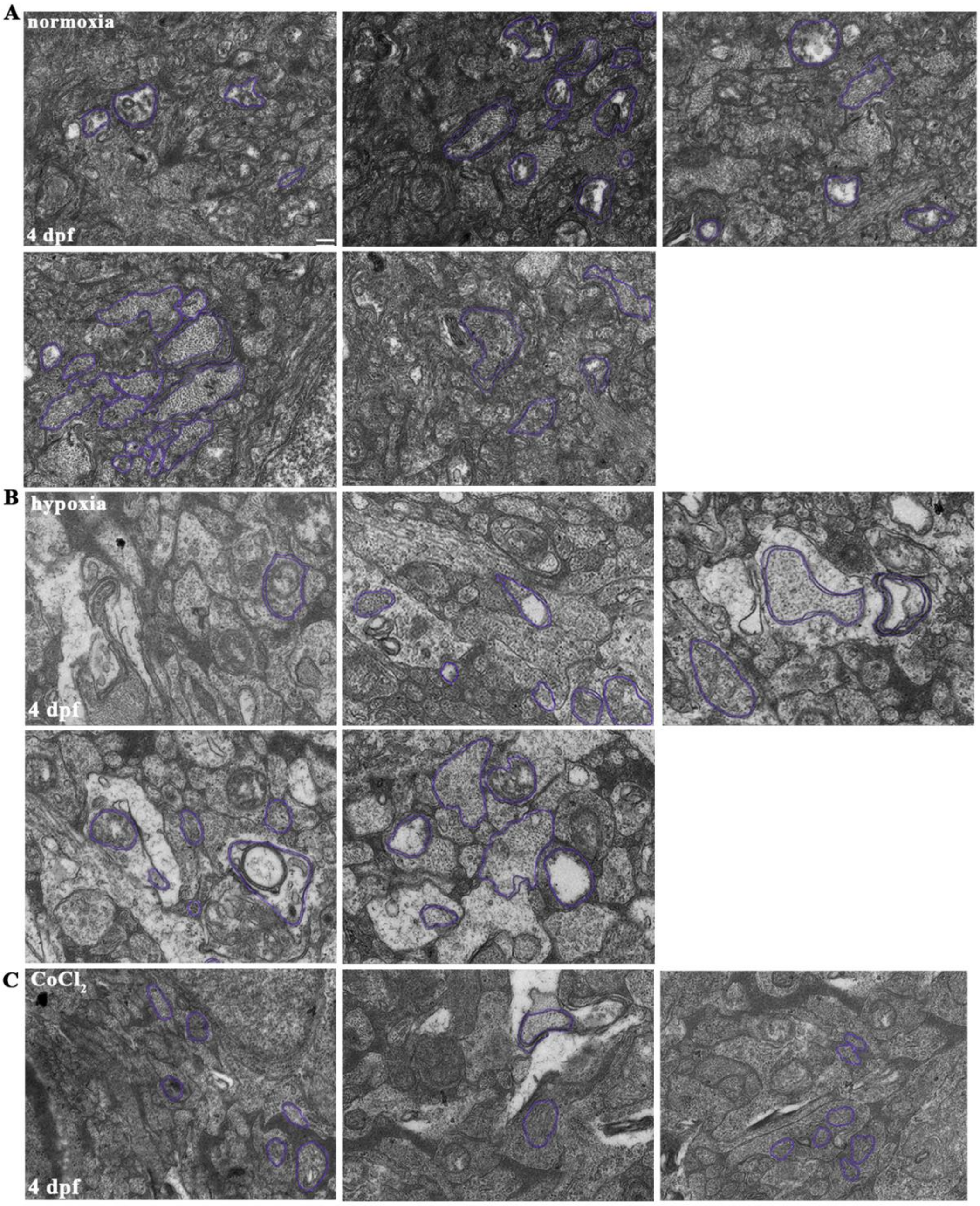

Supplementary Figure 2. The TEM samples for G-ratio analysis and circumference measurement. **(A–C)** The electron micrograph scans of myelinated axons in normoxia, hypoxia, and $CoCl_2$ zebrafish larvae at 4 dpf. The blue inner outlines indicate the axonal circumferences and the blue outer outlines indicate the total (axon and myelin sheath) circumferences. A G-ratio was calculated for the myelinated axons and it was equal to the factor of axon circumference against total circumference. Scale bar: 200 nm.

1.3 Supplementary Figure 3

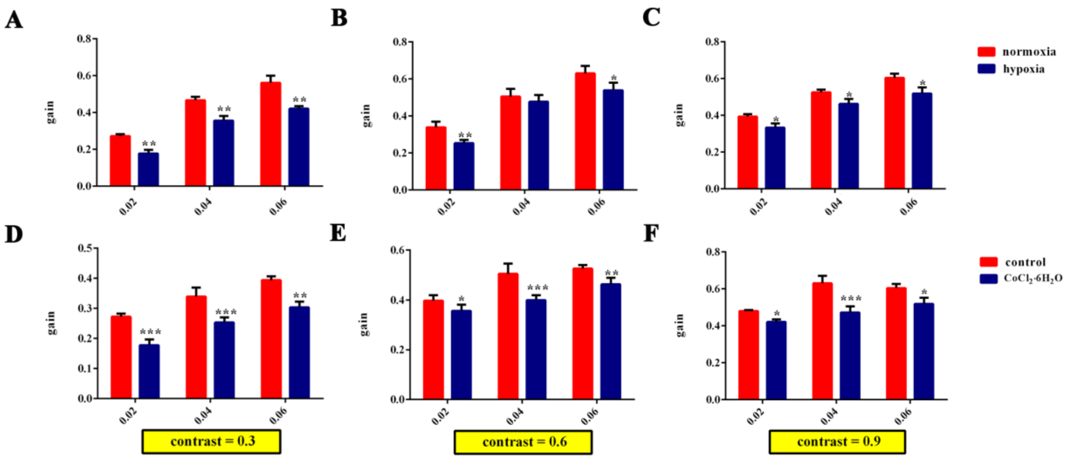

Supplementary Figure 3. Hypoxia resulted in OKR deficiency in larval zebrafish. **(A–C)** OKR behavior tests of normoxia and hypoxia zebrafish larvae at 5 dpf were completed under different cycle/degree and contrast conditions. **(A)** Two-way ANOVA, $P < 0.0001$: nonparametric tests, $^*P < 0.05$ on cycle/degree 0.02; $^{**}P < 0.01$ on cycle/degree 0.04; $^{**}P < 0.01$ on cycle/degree 0.06; n = 10 for each group. **(B)** Two-way ANOVA, $P = 0.0319$: nonparametric tests, $^{**}P < 0.01$ on cycle/degree 0.02; $P = 0.0656$ cycle/degree 0.04; $^*P < 0.05$ cycle/degree 0.06; n = 10 for each group. **(C)** Two-way ANOVA, $P = 0.0002$: nonparametric tests, $^{**}P < 0.01$ on cycle/degree 0.02; $P = 0.0540$ on cycle/degree 0.04; $P = 0.0547$ on cycle/degree 0.06; n = 10 for each group. **(D–F)** OKR behavior tests of control and $CoCl_2$ groups were performed under different cycle/degree and contrast conditions. **(D)** Two-way ANOVA, $P < 0.0001$: nonparametric tests, $^{**}P < 0.01$ on cycle/degree 0.02; $P = 0.0547$ on cycle/degree 0.04; $^{**}P < 0.01$ on cycle/degree 0.06; n = 10 for each group. **(E)** Two-way ANOVA, $P = 0.0003$: nonparametric tests, $P = 0.2324$ on cycle/degree 0.02; $^{**}P < 0.01$ cycle/degree 0.04; $P = 0.2031$ cycle/degree 0.06; n = 10 for each group. **(F)** Two-way ANOVA, $P < 0.0001$: nonparametric tests, $^*P < 0.05$ on cycle/degree 0.02; $^*P < 0.05$ on cycle/degree 0.04; $P = 0.0547$ on cycle/degree 0.06; n = 10 for each group. $^*P < 0.05$, $^{**}P < 0.01$, $^{***}P < 0.001$. Error bars represent S.E.M.

Table 1--Plasmid constructs primer sequences

Gene	Note	Forward primer(5’-3’)	Reverse primer(5’-3’)
mbp	qRT-PCR	CCGTCGTGGAGACGTCAA	CGAGGAGAGGACACAAAGC
olig2	qRT-PCR	ATCCGTCCAGTTGTGGCACT	TGGTGGAAGCAGAGGATGGT
bmp2b	qRT-PCR	TCAGTGATGTCGGCTGGAAC	TGGCATGGTTGGTGGAGTTT
β-actin	qRT-PC	CCCTGTTCCAGCCATCCTT	TTGAAAGTGGTCTCGTGGATACC
bmp2b	R	ATGGTCGCCGTGGTCC	TCGGCAACCGCAGCC

	PCR
